# ﻿A new genus of minute stingless bees from Southeast Asia (Hymenoptera, Apidae)

**DOI:** 10.3897/zookeys.1089.78000

**Published:** 2022-03-16

**Authors:** Michael S. Engel, Lien Thi Phuong Nguyen, Ngat Thi Tran, Tuan Anh Truong, Andrés F. Herrera Motta

**Affiliations:** 1 Division of Entomology, Natural History Museum, University of Kansas, 1501 Crestline Drive – Suite 140, Lawrence, Kansas 66045, USA; 2 Department of Ecology & Evolutionary Biology, University of Kansas, Lawrence, Kansas 66045, USA; 3 Division of Invertebrate Zoölogy, American Museum of Natural History, Central Park West at 79; 4 th; 5 Street, New York, New York 10024-5192, USA; 6 Graduate University of Science and Technology, Vietnam Academy of Science & Technology, 18 Hoang Quoc Viet Road, Nghia Do, Cau Giay, Hanoi, Vietnam; 7 Insect Ecology Department, Institute of Ecology & Biological Resources (IEBR), Vietnam Academy of Science & Technology, 18 Hoang Quoc Viet Road, Nghia Do, Cau Giay, Hanoi, Vietnam; 8 Bee Research Centre, National Institute of Animal Sciences, 9 Tan Phong, Thuy Phuong, Bac Tu Liem, Hanoi, Vietnam

**Keywords:** Apoidea, *Lisotrigona*, Meliponini, taxonomy, Vietnam

## Abstract

A new genus of minute stingless bees (Meliponini: Hypotrigonina) is described from Southeast Asia. *Ebaiotrigona* Engel & Nguyen, **gen. nov.**, is based on the type species *Lisotrigonacarpenteri* Engel, recorded from Vietnam, Thailand, Laos, Cambodia, and southern China. The species was previously considered an enigmatic member of *Lisotrigona* Moure, but is removed to a new genus based on its unique male terminalia that differs considerably from that of *Lisotrigona* and instead shares resemblances with *Austroplebeia* Moure, and the presence of yellow maculation (also similar to that of *Austroplebeia*). It is possible that *Ebaiotrigona* is the extant sister group of *Austroplebeia*, but this requires confirmation by future phylogenetic analyses. Based on available biological observations, *Ebaiotrigonacarpenteri* could not be confirmed as lachryphagous as is well documented from the tear-drinking species of *Lisotrigona* and *Pariotrigona* Moure.

## Abstract (vi)

Một giống mới của ong không ngòi đốt (Meliponini: Hypotrigonina) được mô tả ở Đông Nam Châu Á, *Ebaiotrigona* Engel & Nguyen, **gen. nov.** dựa trên loài chuẩn *Lisotrigonacarpenteri* Engel, được ghi nhận ở Việt Nam, Thái Lan, Lào, Cămpuchia và miền nam Trung Quốc. Loài này trước đây được coi là một thành viên bí ẩn của giống *Lisotrigona* Moure, nhưng đã được chuyển sang giống mới dựa trên các đặc điểm độc nhất của bộ phận sinh dục có sự khác biệt đáng kể so với các loài khác của giống *Lisotrigona* và thay vào đó có những điểm tương đồng với giống *Austroplebeia* Moure, cùng với sự hiện diện của các đốm màu vàng trên cơ thể (cũng tương tự như ở giống *Austroplebeia*). Có thể giống *Ebaiotrigona* có mối quan hệ gần gũi với giống *Austroplebeia*, nhưng điều này cần được chứng minh bằng các phân tích về phát sinh loài trong tương lai. Dựa trên các quan sát về sinh học hiện có, *Ebaiotrigonacarpenteri* chưa thể được khẳng định là một trong những loài uống nước mắt (lachyrphagous) như đã được ghi nhận rõ ràng về tập tính của các loài thích uống nước mắt ở hai giống *Lisotrigona* và *Pariotrigona* Moure.

## ﻿Introduction

Among the considerable diversity of stingless bees (Meliponini), several species stand out for their Lilliputian sizes, typically with body sizes under 4.1 mm. Concomitant with these minute proportions is the further reduction of the wing venation beyond that of most meliponines, lacking closed cells in the hind wing, lacking defined submarginal cells, and the disappearance of 2Rs+M or 3M without a bend apically (Michener 2001). Such diminutive bees occur independently in various lineages of Meliponini and can be found in the genera *Austroplebeia* Moure, *Plebeia* Schwarz, *Proplebeia* Michener, *Scaura* Schwarz, and *Tetragonula* Moure. In addition, ten genera include exclusively tiny species: *Asperplebeia* Engel, *Exebotrigona* Engel & Michener, *Friesella* Moure, *Hypotrigona* Cockerell, *Kelneriapis* Sakagami, *Liotrigona* Moure (sensu Engel et al. 2021), *Liotrigonopsis* Engel, *Lisotrigona* Moure, *Pariotrigona* Moure, and *Trigonisca* Moure (Michener 2001). Michener (2001) discussed the various features of minute Meliponini as well as traits that indicate the relationship, or lack of a close relationship, among the various groups, and considered some genera of this group as synonyms of others (e.g., *Friesella* as a synonym of *Plebeia*, *Tetragonula* as a synonym of *Heterotrigona* Schwarz in a polyphyletic *Trigona* Jurine).

During a revision of *Lisotrigona* (Engel 2000), one species stood out as remarkably distinct from its congeners. *Lisotrigonacarpenteri* Engel, based on a type series collected in northern Vietnam, was unique among all other members of the genus for the presence of yellow maculation on the face, pronotal lobe, and sometimes the mesoscutellar apex. At that time, males for any species of the genus were unknown and *L.carpenteri* was interpreted to be an autapomorphic taxon based on these morphological features of the worker caste. Subsequently, males of *L.furva* Engel (Michener 2007a) and *L.cacciae* (Nurse) (under two synonymic names: Viraktamath and Sajan Jose 2017) were discovered, revealing a unique and remarkably distinctive morphology, further emphasizing the distinctiveness of *Lisotrigona* from other minute genera, and particularly its relatives in Asia and Australia, *Pariotrigona* and *Austroplebeia*. Moreover, it was also discovered that *Lisotrigona* and *Pariotrigona* were lachryphagous, collecting tears from the eyes of various vertebrates (Bänziger et al. 2009, 2011; Bänziger and Bänziger 2010; Bänziger 2018). While these significant revelations were brought forward, the biology and males of *L.carpenteri* remained elusive.

Recently, two groups have independently discovered males of *L.carpenteri* and noted the considerable morphological departure in the characters of the terminalia from other *Lisotrigona* (Li et al. 2021; herein). In fact, the terminalia are generally more similar to that of *Austroplebeia* than to any other minute Meliponini, and certainly lack the synapomorphies that otherwise characterize *Lisotrigona* (Michener 2007a). The male terminalia of *L.carpenteri* are so remarkably different from *Lisotrigona* that the species is here considered to belong to a separate genus. Here we provide a description of the genus and the male, and encourage melittologists to seek additional nests, immature stages, and further biological data for this unique species among the Southeast Asian bee fauna.

## ﻿Materials and methods

**Figure 1.**
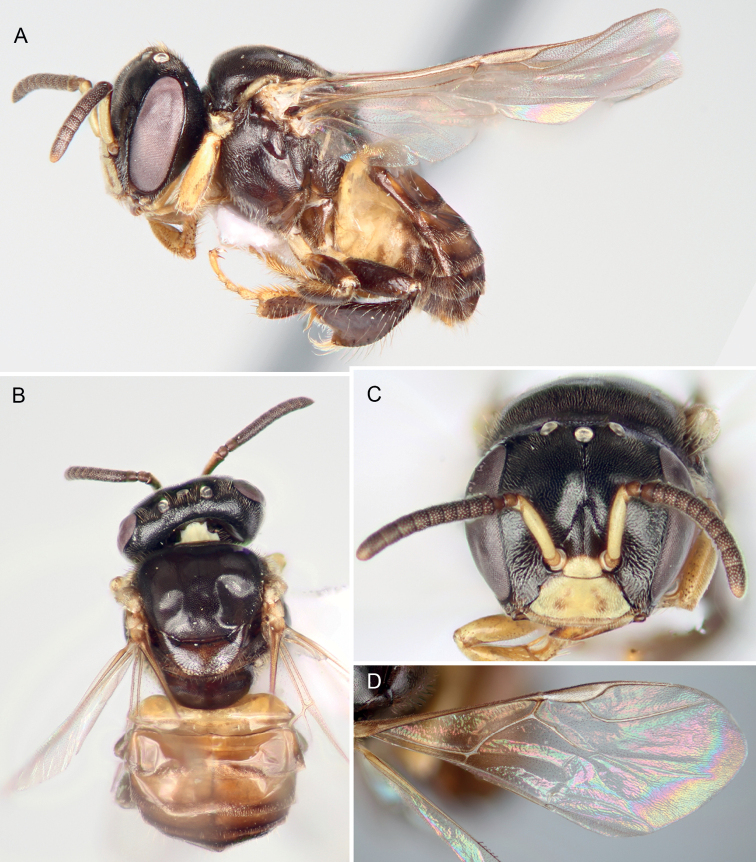
Worker of *Ebaiotrigonacarpenteri* (Engel), comb. nov., light morph **A** lateral habitus **B** dorsal habitus **C** facial view **D** forewing.

Material of *L.carpenteri* was examined from the following collections: Division of Entomology, University of Kansas Natural History Museum, Kansas (**SEMC**); Division of Invertebrate Zoölogy, American Museum of Natural History, New York (**AMNH**); and Insect Ecology Department, Institute of Ecology & Biological Resources (**IEBR**), Hanoi, Vietnam. Morphological terminology follows that of Engel (2001, 2019), Michener (2007b), and Engel et al. (2021). The format for the generic description follows that of Rasmussen et al. (2017), Engel (2019), and Engel et al. (2021). Bees were sampled from sites with a series of nests and swept by net from around the nesting area. Most nests were left undisturbed, although two were exposed but contents were not sampled. Figs 1, 4 were prepared at the University of Kansas, while the remainder were prepared by the Institute of Ecology & Biological Resources.

## ﻿Systematics

### ﻿ Ebaiotrigona

Taxon classificationAnimaliaHymenopteraApidae

Engel & Nguyen gen. nov.

C798E650-7253-50E4-B47E-6C63F1E040F3

http://zoobank.org/99CCDC03-E122-46B9-81F6-D8C11E432DA2

#### Type species.

*Lisotrigonacarpenteri* Engel, 2000.

#### Diagnosis.

The new genus resembles both *Lisotrigona* and the smallest individuals of *Austroplebeia*. The presence of yellow maculation on the face and mesosoma of the worker differentiates this caste from those of *Lisotrigona* (Figs 1, 5–7), while in males the clypeus can be brownish to black (refer to Description, vide). In addition, workers of *Lisotrigona* have distinct erect bristles on the antennal scape (lacking in *Ebaiotrigona*) and have minute plumose facial setae covering the entire face, including the upper frons (in *Ebaiotrigona* the upper frons is lacking in plumose setae and instead covered by minute, fine, simple setae). While yellow maculation is shared between workers of *Ebaiotrigona* and *Austroplebeia*, the latter has a more complete wing venation (Fig. 1D for *Ebaiotrigona* venation), with 1Cu, 2Cu, and 3Cu present, at least as nebulous if not tubular veins, and thereby a more completely delimited subdiscoidal (= second cubital) cell. Additionally, *Austroplebeias.str.* has 6 distal hamuli (versus 5 in *Ebaiotrigona*, *Lisotrigona*, and *Anteplebeina*), and *Anteplebeina* has 1M and 1cu-a confluent (versus 1M distad 1cu-a in *Lisotrigona* and *Austroplebeias.str.*). Perhaps the most dramatic differences between *Ebaiotrigona* and other minute stingless bees are in the male terminalia. The male terminalia of *Ebaiotrigona* (Fig. 4) lack those distinctive features of *Lisotrigona*, that is, in the latter the gonocoxae are enormously expanded proximally into an incomplete ring, the slender gonostyli with apical setae are articulated at about midlength on the elongate gonocoxae, the genital capsule is schizogonal, and sterna VI and VII are entirely different (cf. Fig. 4 vs figs 4, 5 of Michener 2007a). Instead, in *Ebaiotrigona* the genital capsule is rectigonal, with more transverse gonocoxae in which the gonostyli articulate more distally. Furthermore, the gonostyli are uniquely modified: flattened laterally, with lamellate margins broadened proximally on both ventral and dorsal sides, and tapering apically to an acute point lacking setae. The bulb of the penis valve is enlarged and longer than broad, and abruptly tapers to a thin apical process that is shorter than the basal bulb. In *Lisotrigona*, the bulb is smaller and about as long as the apical process, the former gently tapering into the latter (Michener 2007a).

#### Description.

⚲: Minute, total length ca 3.95–4.15 mm, forewing length ca 2.96–3.10 mm; integument generally smooth and polished, some places with widely scattered minute punctures on head and mesosoma, with distinct pale yellow maculation on face, specifically undersurface of scape, supraclypeal area, and clypeus; pronotal lobe; and sometimes as small triangle on lower parocular area, as thin line on lateral margin of mesoscutum bordering tegula and as apicolateral spots on mesoscutellar apical margin (such yellow maculation absent in *Lisotrigona* and *Pariotrigona*, present but more extensive in *Austroplebeia*). Setae generally pale to white; those of body fine, short, and simple, face with minutely plumose setae except on upper frons with only minute simple setae (in *Lisotrigona* the minutely plumose setae distributed across face, including upper frons), scape with fine minute simple setae but without erect short bristles (erect short bristles present in *Lisotrigona*); mesoscutum with numerous erect, fine, short, simple setae; disc of mesoscutellum with similar setae to those of mesoscutum except twice as long or longer, particularly along posterior margin.

Head as broad as mesosoma, slightly broader than long, with face narrower than compound eye length; vertex gently rounded, not produced or ridged; preoccipital area rounded; scape shorter than antennal-ocellar distance, not reaching median ocellus; ocelli near top of vertex; flagellomere I trapezoidal, longer than flagellomere II; flagellomere II about as long as flagellomere III, each slightly broader than long; middle flagellomeres about as long as or slightly longer than broad; intertorular distance slightly shorter than torulocular distance; upper torular tangent below facial midlength; gena rounded, narrower than compound eye in profile; supraäntennal area with triangular medial elevation bordered laterally by converging furrows forming distinct scapal basins; frontal carina absent, indicated on supraäntennal triangle by weakly demarcated ridge, otherwise a narrowly polished line to median ocellus; malar area nearly linear, subequal to or shorter than 0.5× flagellar diameter (similar to *Lisotrigona* and *Austroplebeias.str.*; *Pariotrigona* with malar space slightly longer than flagellar diameter; *Anteplebeina* with malar space as long as flagellar diameter); labrum transverse, simple, apical margin rounded; mandible with apical margin slightly oblique, largely edentate except for two small teeth on upper margin of margin (thus, bidentate), teeth well defined, acute, and incised, interdental spaces distinct but not broadly incised; labial palpomeres I and II with a few, scattered, elongate, apically arched to slightly wavy setae, such setae more clustered apically and sparse elsewhere (similar to that of *Lisotrigona*).

Mesoscutum with median sulcus faintly impressed, extending to slightly beyond mesoscutal midlength; notauli scarcely evident, not impressed; mesoscutellum short, acutely rounded in lateral aspect, overhanging metanotum and extreme base of propodeum, shining transverse depression on mesoscuto-mesoscutellar sulcus simple. Propodeum gently sloping; basal area slightly longer than mesoscutellum, shining, finely and faintly tessellate; propodeal spiracle elongate, nearly meeting metapostnotal lateral arms.

Forewing extending beyond apex of metasoma, with 2Rs, 1rs-m, 1m-cu, 3M, 4M, 1Cu, 2Cu, 3Cu, and 2cu-a absent or at most indicated by spectral traces; membrane hyaline, clear, with weak iridescent reflections apically; prestigma short, subequal to anterior width of 1Rs; pterostigma slender, subparallel margins slightly widening distally to point of 1r-rs, then arching to anterior margin along marginal cell base; marginal cell separated from wing apex by more than its maximum width, apex broadly open, opening about 0.75× maximum marginal cell width, 4Rs trace not angled apically (i.e., not appendiculate); 1M distad 1cu-a (thus, minute 2M+Cu present); submarginal slightly acute; 1M weakly arched; 2M not angled apically (i.e., angle between 2M and spectral 3M linear or at most faintly less than 180°); r-rs slightly longer than 3Rs. Hind wing with 5 distal hamuli; no closed cells and veins posterior to R absent except proximal arch of M+Cu.

Metatibia approximately triangular, approximately 2.8–3.0× as long as greatest width; retromargin gently curved with subangulate distal superior angle, retromarginal setae and superior prolateral surface setae simple; prolateral surface shallowly concave apically, with corbicula occupying slightly less than apical half; retrolateral surface with broad keirotrichiate zone and narrow superior subglabrous zone, without defined clivulus except proximally; keirotrichiate zone approximately 4× as broad as superior glabrate zone, keirotrichiate zone extending to apical margin, with keirotrichiate zone narrowing and superior glabrate zone broadening in apical-most portion of metatibia; inferior penicillum and rastellar comb present, each composed of fine soft setae. Metabasitarsus with proventral margin straight, retrodorsal margin generally paralleling proventral margin, apical margin weakly convex to comparatively straight, distal superior angle not projecting; retrolateral surface without basal sericeous area.

Metasoma subtriangular, about as wide as mesosoma, with metasomal terga smooth and shining except apical margins more matte and faintly and minutely imbricate, all terga almost glabrous, except by minute, erect, simple setae apically forming a narrow band on apical margin, such setae progressively longer, more abundant, and more spread on apical-most terga; sterna largely smooth and glabrous, with long to elongate, fine, erect, simple setae apically.

**Figure 2.**
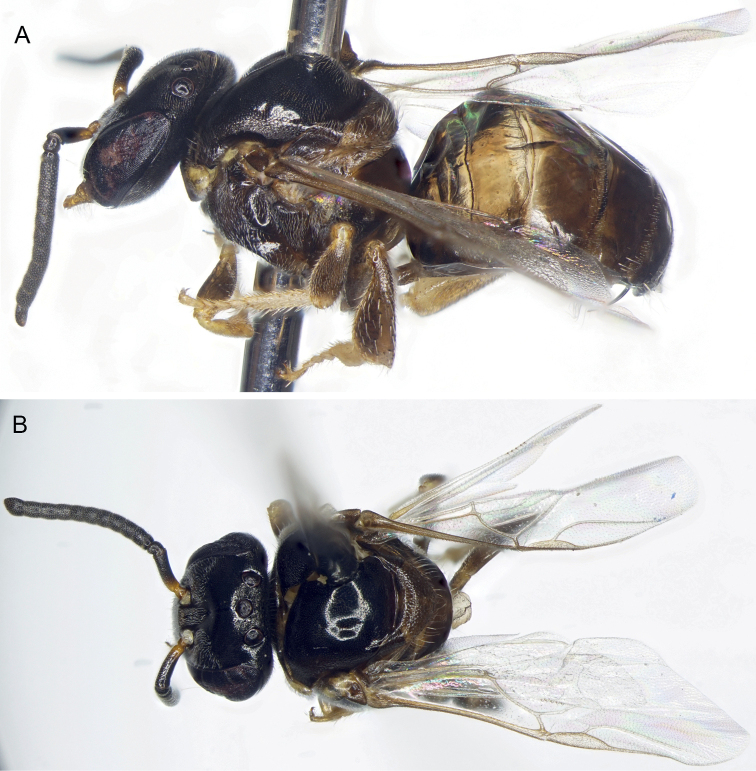
Drone of *Ebaiotrigonacarpenteri* (Engel), comb. nov. **A** lateral habitus **B** dorsal habitus.

**Figure 3.**
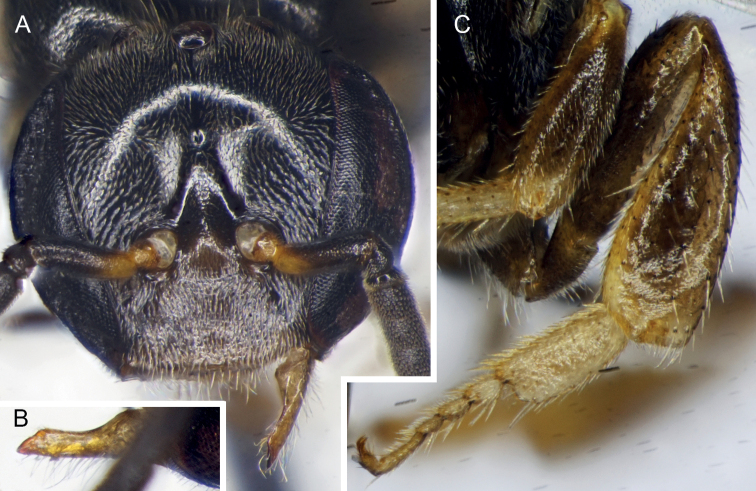
Drone of *Ebaiotrigonacarpenteri* (Engel), comb. nov. **A** facial view **B** outer view of mandible **C** metatibia and metatarsus.

**Figure 4.**
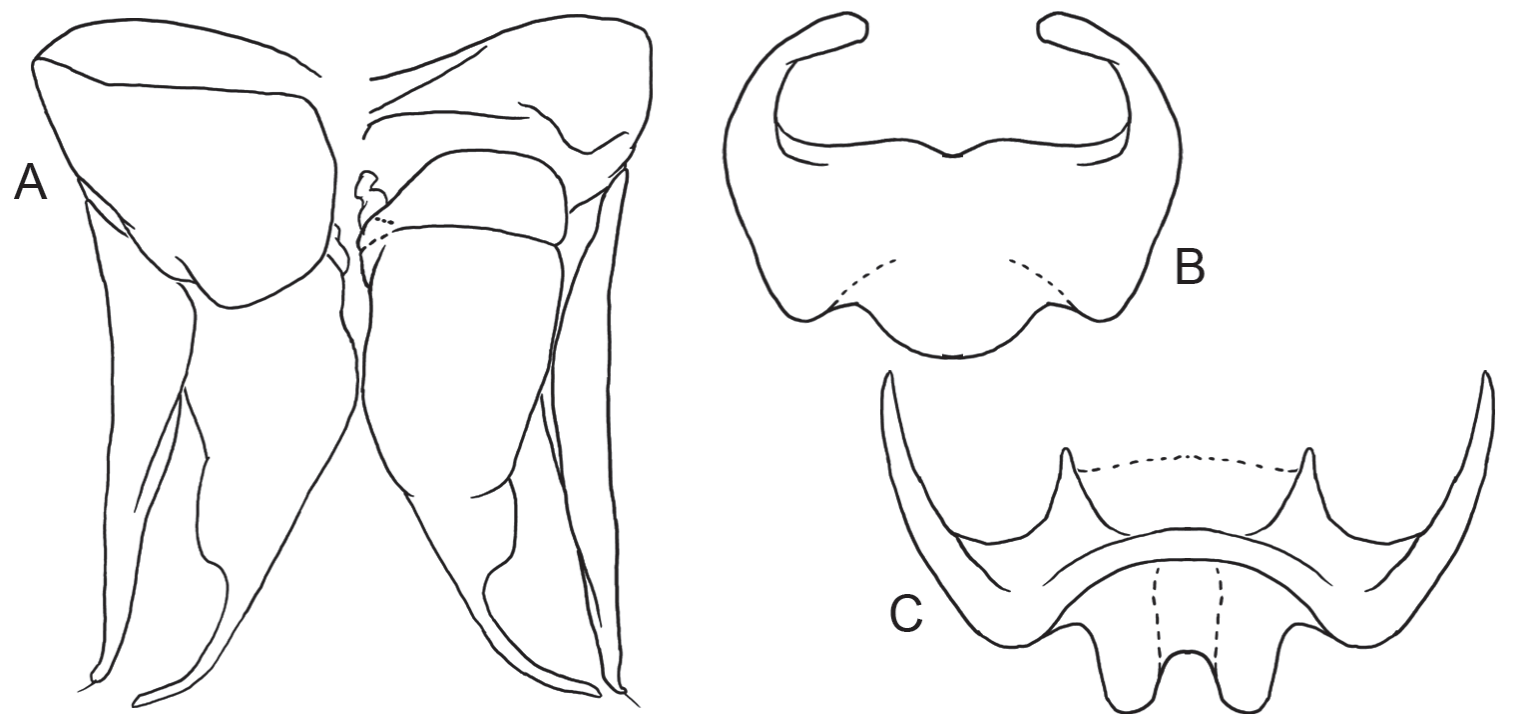
Male terminalia of *Ebaiotrigonacarpenteri* (Engel), comb. nov. **A** genital capsule (left = dorsal, right = ventral) **B** metasomal sternum VII **C** sternum VI.

♂: Darker than worker, apparently without facial maculation of worker (Figs 2, 3), instead yellow areas of worker dark brown to black in drone [brown in Vietnamese male, wholly black in Chinese male (Li Yuran, pers. comm. and unpublished images to MSE)] [note that sometimes areas of yellow maculation start off brownish in individuals who are not fully pigmented and so brown areas of the male could eventually become fully yellow as in the worker; however, the fact that the Vietnamese male appears to be fully pigmented elsewhere on the body (Figs 2, 3) and that the Chinese male is wholly black tends to suggest that drones of *Ebaiotrigona* truly lack facial maculation, but this will require confirmation through extensive future sampling of males throughout the range of the species], except pronotal lobe consistently yellow. Scape shorter and broader than that of worker (Fig. 3); flagellomere I trapezoidal, shorter than pedicel or flagellomere II; flagellomere II about as long as flagellomere III, each slightly longer than broad. Metasomal sternum VI medioapically chamfered, bilobed; sternum VII medially broadly convex and slightly depressed, with shallow apicolateral concavities; genital capsule rectigonal (Fig. 4); gonobase somewhat transverse; gonocoxae broader than long, with gonostylus articulating somewhat proximally; gonostylus elongate and bladelike, flattened laterally, and broadened proximally and lamelliform mesally on dorsal and ventral sides, tapering apically to acute point with a single fine apical seta, seta simple (Fig. 4); penis valves elongate, bulb enlarged, longer than broad, well sclerotized, abruptly tapering to thin elongate apical process, process gently arched and acutely rounded apically, process slightly shorter than bulb, blub without elongate proximal apodeme, instead with short, twisted apodeme (Fig. 4).

#### Etymology.

The new generic name is a combination of the Ancient Greek adjective *ēbaiós* (*ἠβαιός*, meaning “small”) and the generic name *Trigona* Jurine. The gender of the name is feminine.

#### Included species.

Currently, the genus includes only the type species, *Ebaiotrigonacarpenteri* (Engel), new combination.

The following modifications to the key of Rasmussen et al. (2017) and Engel et al. (2018) allow for the incorporation of *Ebaiotrigona*.

**Table:** 

1	Forewing length less than 3.2 mm, wing venation greatly reduced and retrodorsal margin of metatibia without plumose setae; hind wing without closed cells, veins closing radial and cubital cells, if visible at all, clear and unpigmented (spectral); forewing with 2Rs and 1rs-m almost always completely absent, thus without indication of submarginal cells; at least distal part of second cubital cell of forewing undefined or defined completely by unpigmented spectral vein traces (i.e., at least 2Cu and 3Cu absent or spectral); vein M of forewing terminating without bend at about position of anterior end of 1m-cu which, however, is absent (i.e., 3M lacking)	**2**
–	Forewing length typically over 4 mm, wing venation typically not greatly reduced for Meliponini, but if minute and with some wing reduction, then retrodorsal margin of metatibia with plumose setae intermixed with simple setae; hind wing typically with radial and cubital cells closed by at least weakly brownish nebulous veins; forewing with one or two submarginal cells usually weakly indicated by nebulous traces of 2Rs and 1rs-m, first submarginal cell usually recognizable; second cubital cell of forewing completely indicated by at least faint nebulous veins (i.e., 2Cu present); vein M of forewing usually extending at least slightly beyond position of 1m-cu and angular at apex of tubular portion of vein (i.e., 3M present), the stub of which is usually at least faintly visible	**3**
2	Malar space shorter than flagellar diameter; inner margins of compound eyes converging below	**2a**
–	Malar space almost one-fifth as long as compound eye, much longer than flagellar diameter; inner margins of compound eyes nearly parallel	***Pariotrigona* Moure**
2a	Yellow maculation present in worker on scape, supraclypeal area, clypeus, pronotal lobe and sometimes on lower parocular area, apically on mesoscutellum, and laterally on mesoscutum; scape without erect bristles; minutely plumose facial setae absent on upper frons; gonocoxae unmodified, with gonostyli articulating more distally; gonostyli elongate, bladelike, expanded and lamellate proximally; genital capsule rectigonal; metasomal sternum VI medioapically chamfered, bilobed	***Ebaiotrigona* , gen. nov.**
–	Yellow maculation lacking, at most with pale yellow brown areas; scape with erect bristles; minutely plumose facial setae extending across upper frons; gonocoxae with enormous, arched, proximal extensions, with gonostyli articulating near midlength; gonostyli slender elongate; genital capsule schizogonal; metasomal sternum VI with a single medioapical process	***Lisotrigona* Moure**

### ﻿ Ebaiotrigona carpenteri

Taxon classificationAnimaliaHymenopteraApidae

(Engel) comb. nov.

575C2D28-D4B0-5DC4-A161-410B5976B64B

**Figure 5.**
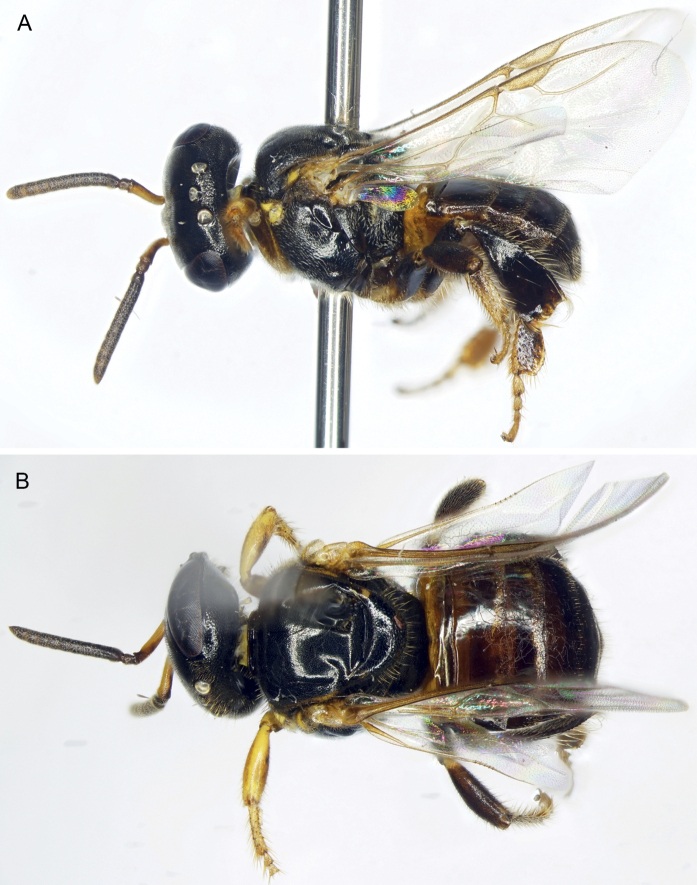
Worker of *Ebaiotrigonacarpenteri* (Engel), comb. nov., dark morph **A** lateral habitus **B** dorsal habitus.

**Figure 6.**
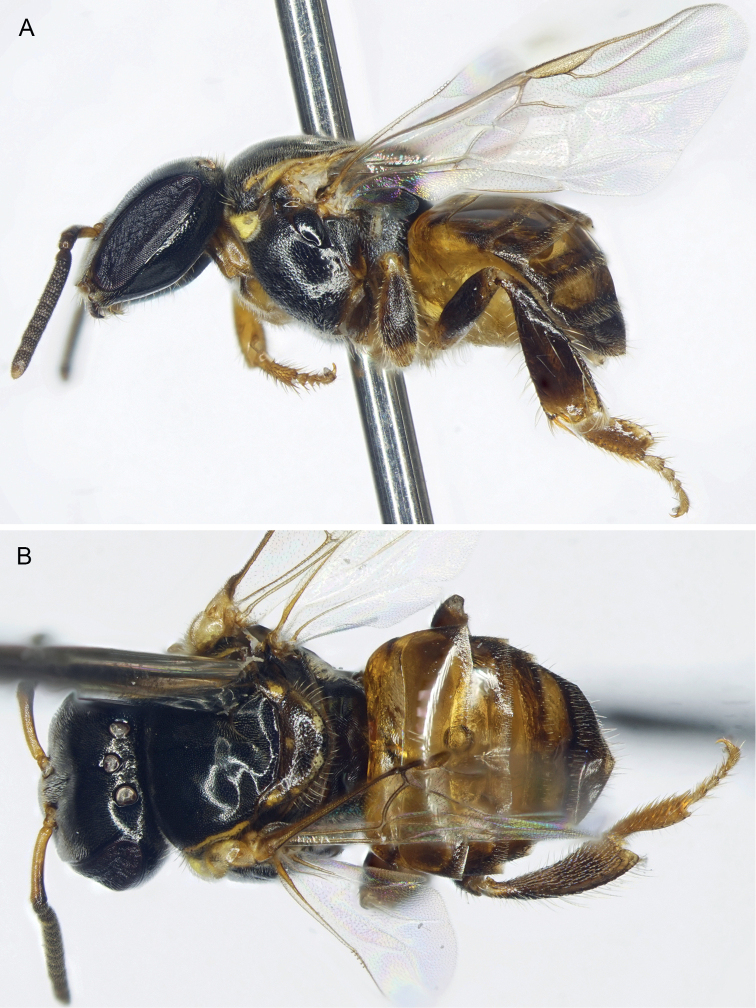
Worker of *Ebaiotrigonacarpenteri* (Engel), comb. nov., intermediate light morph **A** lateral habitus **B** dorsal habitus.

**Figure 7.**
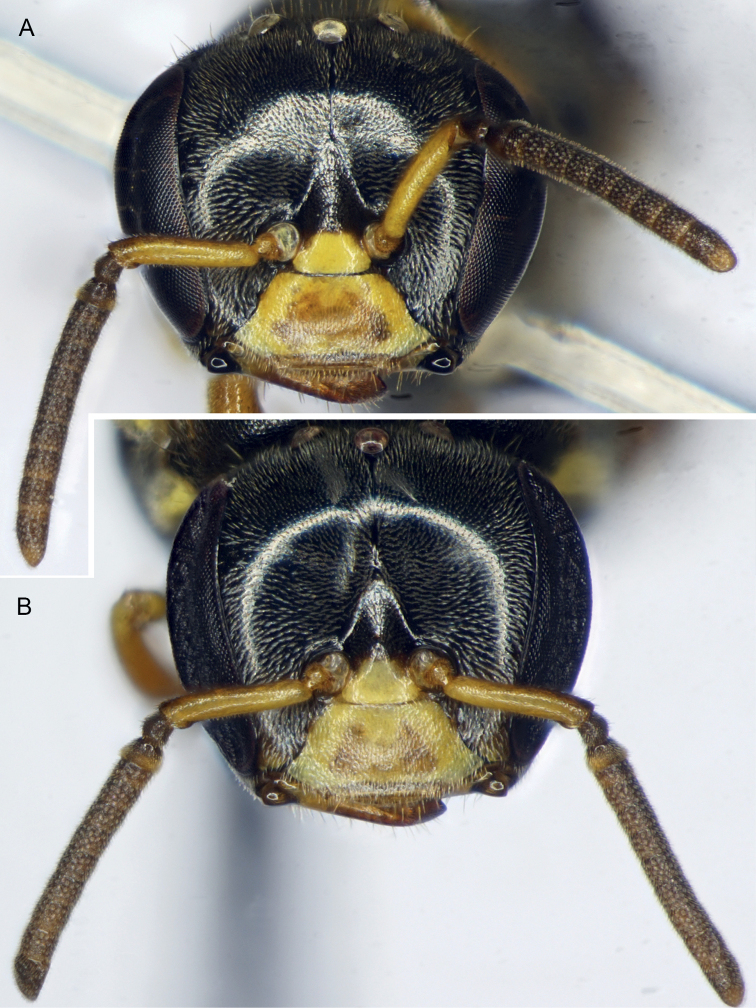
Worker faces of *Ebaiotrigonacarpenteri* (Engel), comb. nov., showing identical patterns in both dark and intermediate light morph **A** dark morph **B** intermediate light morph.

Figs 1 , 2 , 3 , 4 , 5 , 6 , 7

Lisotrigona carpenteri Engel, 2000: 232. Holotype ⚲ (visum, AMNH).

#### Remark.

The worker of this species has been described by Engel (2000). We do not repeat that material here but instead provide descriptive details for the hitherto undocumented male.

#### Descriptive notes.

♂: Body length 3.5 mm; forewing length 2.9 mm; head width 1.29 mm; head length (lower margin of clypeus to anterior margin of median ocellus and to summit of head) 1.04 mm, 1.19 mm, respectively; compound eye length, upper and lower interorbital distances 0.92 mm, 0.53 mm, 0.45 mm, respectively.

Compound strongly converging below, inner orbits only feebly concave above; compound eye length greater than upper interocular distance; antennae arising below lower third of face so that distance from lower clypeal margin to lower margin of antennal torulus is about one-half distance from upper margin of torulus to lower margin of median ocellus; clypeus about twice as broad as long, lower margin scarcely below lower ocular tangent, upper margin separated from antennal torulus by about one-half torular diameter; head surface in vicinity of antennal toruli depressed, not visible in profile; interocellar distance over twice ocellocular distance, almost 3× ocellar diameter; ocelli on submit of vertex, head surface declivitous immediately behind lateral ocelli with no ridge or carina; genal area much narrower than compound eye in profile; malar area linear (mandibular base almost in contact with lower compound eye margin); mandibles comparatively long, conspicuously crossing one another, mandibular apex slender, apical margin oblique with lower apical angle pointed, upper portion of apical margin faintly sinuous and simulating a faint preapical bump (scarcely large enough to denote as a “tooth”) (Fig. 3B); antennal scape short, its length less than half distance from antennal torulus to median ocellus; flagellum slightly tapering, covered with short, dense setae; flagellomere I shorter than pedicel, nearly 3× as broad as long; flagellomere II about 1.25× as broad as long, flagellomere III about as broad as long, subsequent flagellomeres progressively shorter so that flagellomeres IV–VI are shorter than broad, flagellomeres VII–X progressively longer, longer than broad, flagellomere XI with broadly rounded apex. Male terminalia as in Fig. 4.

Integument of head and mesosoma black except basal one-third of scape (grading into black distal half), mandible, labrum, clypeus, spot between antennae, mesoscutellum, metanotum, propodeum, legs except coxae and femora, and all metasomal segments, brown or suffused with brown ranging to black (darkest in Chinese male: Li Yuran, pers. comm. and image to MSE); pronotal lobe markedly yellow; protibia, meso- and metatibial apices, and tarsi light yellowish brown; tegulae yellowish brown; wings clear, veins light brown; stigma translucent, light brownish.

Integument shining, smooth, head and mesosoma with well separated, fine, small punctures, punctures on lateral part of mesoscutum denser; punctures on mesoscutellum much stronger and denser than those on mesoscutum, separated by 2–3× a puncture width; mesepisternum centrally with coarse punctures separated by 2–3× a puncture width, otherwise integument between punctures smooth; basal area of propodeum shining, minutely tessellate, glabrous; posterior surface smooth, shining. Metasomal terga smooth except minute punctures on narrow posterior margins.

Pubescence mostly exceptionally short, mostly yellowish white, setae of lower half of face white and conspicuously plumose, other setae simple or nearly so. Posterior part of mesoscutellum with zone of upcurved setae, longest on body except for some on apical margin of clypeus and mandible, about 2.5× as long as median ocellar diameter, otherwise long setae (nearly 2× ocellar diameter) sparse on apical margin of clypeus, vertex, coxae, and trochanters, rather numerous on mandibular lower margin. Setae of mesoscutum short, some setae at apicolateral corner longer, about as long as ocellar diameter. Metasomal terga I–IV glabrous except for minute erect setae on posterior margins.

#### Variation.

Workers exhibit noticeable variation in overall coloration, and can loosely fall into a lighter and dark morph, although there are individuals who seemingly intergrade and so these morphs are not discrete (Figs 1, 5–7). Generally, areas of maculation on the clypeus, ventral surface of scape, mesoscutal lateral borders, axilla, apicolaterally on mesoscutellum, foreleg, and in isolated areas on the mid- and hind legs can vary from pale to vivid yellow, while the males the tarsi are more consistently yellowish brown to yellow and the podites basal to the tarsi are brown to black (darkest in the Chinese male: Li Yuran, pers. comm. and image to MSE). In addition, the width of the marks on the mesoscutum and mesoscutellum can be exceptionally narrow (mesoscutum) or faint (mesoscutellum), such that they can appear superficially absent. Most noticeably, areas of black on the mesosoma and legs can vary to dark brown (e.g., cf. Figs 1, 5, 6). The metasoma can range from being largely black to dark reddish brown, with lighter brown on the first tergum and basal sterna, to the same pattern but from pallid yellow on the basal sterna and first tergum, with ferruginous on the remaining terga, but blending from light proximally to dark apically, and within a given tergum darkening slightly toward each marginal zone. It should be noted that the holotype worker (AMNH) is of the darker morph.

#### New material examined.

Vietnam: 1♂, 67⚲⚲, Tân Thành [Village], Yên Thịnh [Community], Yên Thủy [District], Hòa Bình [Province] [20°21.35'N, 105°39.47'E], 22 June 2021, coll. Tuấn Anh Trương et al. [1♂, 6⚲⚲, nest #1; 5⚲⚲, nest #2; 5⚲⚲, nest #3; 3⚲⚲, nest #4; 2⚲⚲, nest #5; 12⚲⚲, nest #7; 8⚲⚲, nest #9; 8⚲⚲, nest #19; 12⚲⚲, nest #16; 6⚲⚲, nest #21] (IEBR); 18⚲⚲, Yên Hân [Community], Chợ Mới [District], Bắc Kạn [Province], 27 June 2021, coll. Tuấn Anh Trương et al. [11⚲⚲, nest #11; 1⚲⚲, nest #18; 6⚲⚲, nest #13] (IEBR); 53⚲⚲, Nam Cường [Community], Chợ Đồn [District], Bắc Kạn [Province], coll. Tuấn Anh Trương et al. (6⚲⚲, nest #9; 16⚲⚲, nest #11; 21⚲⚲, nest #21); 21⚲⚲, Lân Nghè, Hữu Liên Natural Reserve, Hữu Liên [District], Lạng Sơn [Province], 21°33'48.6"N, 106°24'36.4"E, ca 289 m, 11 June 2018, coll. Liên Thị Phương Nguyễn et al. (IEBR).

Dark morph: 23⚲⚲, Vũ Quang National Park, Vũ Quang [District], Hà Tĩnh [Province], 18°17'44"N, 105°22'29"E, 12 December 2020, coll. Ngát Thị Trần & Cường Quang Nguyễn (IEBR).

#### Comments.

**Figure 8.**
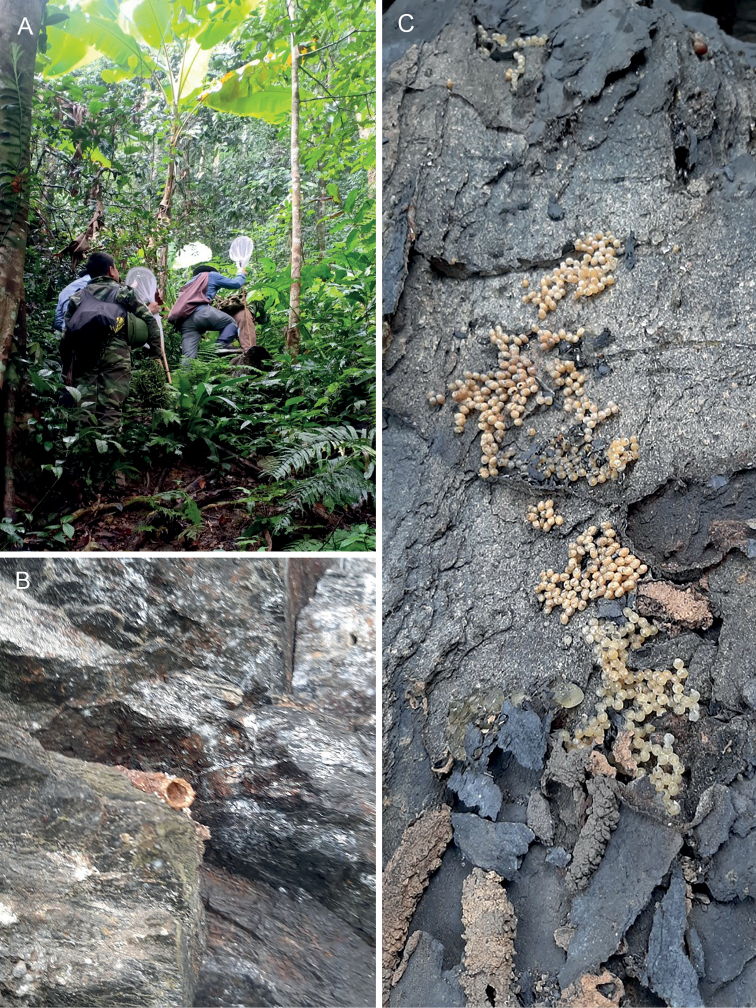
Nests and habitat of *Ebaiotrigonacarpenteri* (Engel), comb. nov., at Bắc Kạn Province, Vietnam **A** general habitat **B** nest entrance in limestone cliff **C** exposed nest from between limestone slabs.

**Figure 9.**
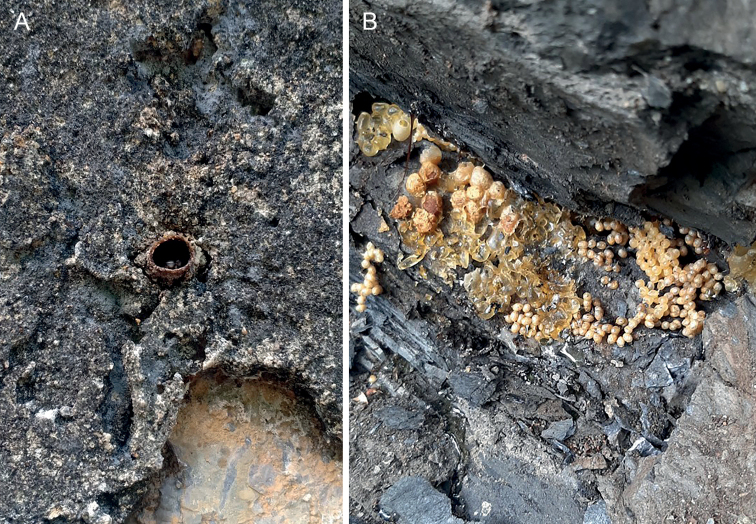
Nests of *Ebaiotrigonacarpenteri* (Engel), comb. nov., at Bắc Kạn Province, Vietnam **A** nest entrance **B** exposed nest from between limestone slabs.

More than 10 nests were observed in a rocky wall in Yên Thủy, Hòa Bình Province, while six or seven were seen in one locality in limestone cliffs in Bắc Kạn Province (Figs 8, 9). The bees appear to prefer nesting in cavities between stones, either natural limestone cliffs or even amid the rocks of human-built walls, much like that of *Pariotrigona* (Bänziger et al. 2011). The bees were quite a nuisance and frequented human skin where they were lapping sweat, and attempted to approach the eyes of the collectors (Truong pers. obs.), although it is unclear if this was to collect tears or merely as a timid form of defense from a perceived threat near the nests. Similar observations on the behavior of the bees were made in southern China (Li et al. in press). It is possible that the bees rely on tears (lachryphagous) in the same manner as *Pariotrigona* and *Lisotrigona*, although no tear collection could be confirmed nor were bees observed visiting the eyes of cattle or other vertebrates. The bees and nests have a strong foul odor, and future work on the chemistry of their nests and stores is needed to determine their composition and the potential derivation of these smells. Future work will more fully explore the nesting biology, nest architecture, and immature stages of *E.carpenteri*.

## ﻿Discussion

While *E.carpenteri* was always a unique species as the only minute stingless bee in Southeast Asia with yellow maculation, it has not been until the discovery of the male that its real distinction was revealed. The male terminalia are clearly not of the structure so typical for *Lisotrigona*. Specifically, *E.carpenteri* lacks the enormous proximal extensions of the gonocoxae unique to species of *Lisotrigona* (Michener 2007a). While this form is wholly unique among Meliponini, the result of the almost proximally ringlike gonocoxae approximates the schizogonal condition as the proximal fossae of the gonocoxae open mesally (Michener 1990). Conversely, in *Ebaiotrigona*, *Pariotrigona*, and *Austroplebeia*, the gonocoxae are not so modified, and the opening of the gonocoxae are directed proximally (Michener 2001; Dollin et al. 2010), and thus of the rectigonal configuration with the sole exception of *Pariotrigona*, which is more amphigonal. In addition, in *Lisotrigona* the gonostyli articulate with the gonocoxae about midlength, whereas in the others, including *Ebaiotrigona*, the gonostyli articulate more distally. Unlike all of the other genera, the gonostyli of *Ebaiotrigona* are broadened proximally as lamellae, elongate, and almost bladelike. This form is unique to the genus.

In several respects *Ebaiotrigona* appears more similar to *Austroplebeia*, and this is borne out by the form of the male genitalia. In both genera the basal bulb of the penis valve is quite enlarged and sclerotized, and tapers apically to an elongate, thin, clawlike process. Note that these extensions in *Austroplebeia* and *Ebaiotrigona*, although well sclerotized and often melanized, are easily broken near their base (e.g., from the photograph of Li et al. (2021: fig. 2d), and in additional images of this male sent to MSE these are broken off in the Chinese male reported therein; nonetheless, the Chinese populations should be further explored in the future as to whether or not they represent a species distinct from *E.carpenteri*). In *Pariotrigona* the basal bulb is smaller and somewhat transverse, and is abruptly narrowed to the thin elongate process. In *Lisotrigona*, the penis valves are comparatively short relative to the overall size of the genitalia, with a broad basal bulb and tapering to a comparatively short process that is scarcely longer than the bulb. While the Asian and Australian minute species lack a spatha, *Pariotrigona* is an outlier in that the spatha is present as a large membranous structure. The form of each of these genera is seemingly apomorphic when compared to putative outgroups among related members of the *Hypotrigona* group of genera (Table 1: sensu Engel et al. 2021). *Liotrigona* has another wholly unique form of genitalia. The capsule is elongate and permanently schizogonal, with the gonocoxae much longer than broad and fused ventrally, while the gonostyli articulate apically (Michener 1990). By comparison, the genitalia of *Hypotrigona* are comparatively drab and more generalized with conditions observed broadly across Meliponini. In *Hypotrigona* the capsule is rectigonal, but apomorphically the opening opens dorsally, while the gonocoxae are short and transverse (Michener 1990). The basal bulb of the penis valve is membranous.

**Table 1.** Asian and Australian species of the *Hypotrigona* group (sensu Engel et al. 2021). Occurrences in brackets are likely but not yet confirmed; data taken from: Ascher et al. (2016), Chinh et al. (2005), Dollin et al. (2015), Engel (2000), Engel et al. (2021), Karunaratne et al. (2017), Le et al. (2021a, 2021b), Lee et al. (2016), Li et al. (in press), Michener (2001, 2007a, 2007b), Nguyen et al. (2021), and Thangjam et al. (in press).

Genus *Austroplebeia* Moure	
Subgenus †*Anteplebeina* Engel	
†*A.fujianica* Engel	China (Fujian) (Miocene)
Subgenus Austroplebeia Moure	
*A.australis* (Friese)	Australia
*A.cassiae* (Cockerell)	Australia
*A.cincta* (Mocsáry)	Indonesia (Papua, West Papua), Papua New Guinea
*A.essingtoni* (Cockerell)	Australia
*A.magna* Dollin et al.	Australia
Genus *Ebaiotrigona*, **gen. nov.**	
*E.carpenteri* (Engel), **comb. nov.**	Cambodia, China*, Laos, Thailand, Vietnam
Genus *Lisotrigona* Moure	
*L.cacciae* (Nurse)	India, Sri Lanka, Thailand, Vietnam**
*L.furva* Engel	Cambodia, [Laos], Thailand, [Vietnam]***
Genus *Pariotrigona* Moure	
*P.pendleburyi* (Schwarz)	Brunei, Cambodia, [Indonesia: Kalimantan], Malaysia, Thailand

* Chinese population may be a separate species of *Ebaiotrigona*, something that is in need of future study, particularly given the habitat differences (Li et al. in press). ** Records of *L.cacciae* from Vietnam by Sakagami (1975: as *Hypotrigonascintillans* (Cockerell)) and Le et al. (2021b) remain to be confirmed. Sakagami (1975) refers to “total melanism”, which could apply to *L.furva*. *** Records of *L.furva* from Vietnam by Le et al. (2021a) remain to be confirmed.

Another unique feature of *Ebaiotrigona* is the shape of the sixth metasomal sternum, which is apically bilobed, versus the single medial process of *Lisotrigona*, *Pariotrigona*, and *Austroplebeia*. The seventh sternum also differs from these genera and therefore highlights the distinctiveness of the new genus among Old World Meliponini.

Given the similar male terminalia and the presence of yellow facial maculation, it leads one to wonder if *Ebaiotrigona* could be the extant sister group of *Austroplebeia*, diverging from this genus prior to the Miocene and when the latter still had a presence in mainland Asia (Engel et al. 2021). Naturally, future phylogenetic analyses of morphological and molecular data need to include a broader sampling of Asian species, and certainly including samples of *E.carpenteri*.

The nests and the biology of *E.carpenteri* remain to be explored in greater detail, work that the authors hope to undertake in the forthcoming years. Limited observations on nests were presented by Chinh et al. (2005), who reported nests in tree trunks, rock crevices, and human-made structures. Chinh et al. (2005) reported amorphous brood clusters, like those observed in here (Figs 8, 9). The contents of the honey pots were not explored. Therefore, for the moment one must wonder whether *E.carpenteri* is actually lachryphagous like *Lisotrigona* and *Pariotrigona*, or a typical floral visitor as in *Austroplebeia*. Certainly current observations suggest that while the species laps sweat, like many bees, *E.carpenteri* may not actually imbibe tears (Li et al. in press; herein). A further elaboration of the nests and biology of *E.carpenteri* will be of considerable significance for understanding the distribution and occurrence of lachyrphagy in Asiatic Meliponini. If true, lachryphagy is restricted to *Lisotrigona* and *Pariotrigona*, then it may be that this is a biological synapomorphy for these two genera, with *Ebaiotrigona* more closely allied to *Austroplebeia*. Ideally, once the biology has been more fully elucidated for *E.carpenteri*, an analysis of morphological and molecular data can be undertaken for the *Hypotrigona* group (Table 1) to explore not only phylogenetic relationships but also patterns of biogeographic and biological significance. Fortunately, a large number of nests are available at localities in northern Vietnam for future investigation.

## Supplementary Material

XML Treatment for Ebaiotrigona

XML Treatment for Ebaiotrigona carpenteri
